# Comparison of Stress and Strain Distribution Patterns in Canine Implant and Maxillary Bone in Three Occlusal Schemes Using Finite Element Analysis

**DOI:** 10.1055/s-0043-1776313

**Published:** 2024-02-08

**Authors:** Amirhossein Fathi, Younes Hoshyar, Behnaz Ebadian, Mahsa Ghorbani

**Affiliations:** 1 Department of Prosthodontics, Dental Material Research Center, Dental Research Institute, Isfahan University of Medical Sciences, Isfahan, Iran; 2 Dental Implants Research Center, Dental Research Institute, Department of Prosthodontics, School of Dentistry, Isfahan University of Medical Sciences, Isfahan, Iran; 3 Dental Students Research Committee, School of Dentistry, Isfahan University of Medical Sciences, Isfahan, Iran; 4 School of Dentistry, Mashhad University of Medical Sciences, Mashhad, Iran

**Keywords:** dental occlusion, dental implants, single tooth, finite element analysis

## Abstract

**Objectives** This study aimed to compare the pattern of stress and strain distribution in canine implant and maxillary bone in the anterior group function (AGF), posterior group function (PGF), and canine guidance (CG) occlusal schemes by finite element analysis (FEA).

**Material and Methods** In this *in vitro* experimental study, a dental implant (10 × 4.1 mm) was inserted at the site of the maxillary canine in a model of the maxilla in Mimics software. The implant was scanned three-dimensionally and the data were transferred to SolidWorks software. The von Mises stress, shear stress, deformation, and strain were calculated in the AGF, PGF, and CG occlusal schemes by FEA.

**Statistical Analysis** Data were analyzed by ABAQUS software to calculate the stress transferred to the canine implant and maxillary bone in the three occlusal schemes.

**Results** The maximum and minimum von Mises stress, elastic strain, shear stress, and deformation were noted in the AGF and PGF occlusal schemes, respectively, in all teeth.

**Conclusion** The PGF showed minimum von Mises stress, elastic strain, shear stress, and deformation in the canine implant and maxillary bone. Thus, it appears than the PGF is the best occlusal scheme for maxillary canine implant followed by the CG scheme.

## Introduction

Use of dental implants has greatly increased for replacement of the lost teeth. They can be used for replacement of one single tooth, multiple teeth, or as an abutment for fixed partial denture or implant-supported overdentures. 1 2 Despite the high success rate reported for single-unit maxillary dental implants, 3 4 they are often associated with mechanical and biological complications. 1 5

It has been reported that some occlusal schemes and lateral movements are associated with greater changes in marginal bone level around dental implants. The reason can be greater occlusal stress distribution in implant fixtures, or the angulation of lateral forces applied to the occlusal surface of implant restorations. 6 7 8 Also, some authors claim that the group function occlusal scheme increases the possibility of contact with the opposing teeth in nonfunctional lateral movements or nonworking side interferences. 6 Nonetheless, several factors affect the magnitude of stress applied to dental implants. The occlusal scheme in lateral movements of the jaw is one such factor, which can be anterior group function (AGF), posterior group function (PGF), or canine guidance (CG).

Finite element analysis (FEA) is an ideal method to analyze the effect of occlusal stress applied to implant components, and evaluate the pattern of stress and strain distribution in dental implant and the adjacent structures. It uses a numerical system to analyze the physical phenomena, load distribution, and behavior of materials in response to load application. While studies have demonstrated that the isotropic bone model can lead to an overestimation of maximum von Mises strain by up to 15% under pure compression and an underestimation of up to 50% under pure torsion when compared with the anisotropic model, 9 it is important to note that the complexities inherent in FEA often necessitate simplifications. In many FEA applications, material properties are commonly assumed to be homogeneous, isotropic, and linearly elastic due to the computational challenges posed by incorporating more complex material behaviors. 10 11 FEA is also ideal to find the points of maximum stress and strain accumulation in tissues.

Considering the gap of information regarding the pattern of stress and strain distribution in maxillary canine dental implants in different occlusal schemes, this study aimed to compare the stress and strain distribution patterns in the canine implant and maxillary bone in the AGF, PGF, and CG occlusal schemes by FEA.

## Material and Methods

In this *in vitro* , experimental study, a dental implant (10 × 4.1 mm) was inserted at the site of maxillary canine in a model of maxilla in Mimics software. To enhance three-dimensional (3D) analysis, the complex was divided into smaller elements. Cone-beam computed tomography images taken with 330-µm voxel size, 61 × 78 mm field of view, high resolution, and scanning time of 12.6 second were used for geometrical reconstruction of cancellous and cortical bone, teeth, and periodontal ligament (PDL) in Mimics software (Materialise Mimics Innovation Suite 21.0). The bones, teeth, PDL, and canine tooth crown were modeled in Mimics and 3-Matic software programs. For this purpose, the cone-beam computed tomography scans with 1-mm slice thickness were transferred to Mimics software. Next, the segmentation tool was used to create masks for the teeth, maxillary bone, and PDL. Then, the “Calculate 3D” command was used to create the 3D model of the components. The data in the STL format were then transferred to Geomagic software and converted to the STP format. Next, the 3D design of a bone-level dental implant (10 × 4.1 mm; Straumann AG, Basel, Switzerland) was scanned by a 3D scanner. The scans along with the data obtained from Geomagic software were transferred to SolidWorks version 5.1 2019 software. SolidWorks was employed for both creating geometric models and refining them during the image reconstruction process from DICOM and 3D scans, before subsequently inputting into FEA software. The software measurement accuracy was 0.001 mm. All materials were considered homogenous and isotropic. The dimensions of the implanted canine tooth were defined according to the values reported in Wheeler's tooth anatomy and morphology software. The final geometry of the components was then transferred from SolidWorks to ABAQUS FEA software. The mechanical properties ( Table 1 ) of the components (modulus of elasticity, Poisson's ratio, and density), 12 13 type of analysis (which was dynamic), interactions between the components (the amount of friction), magnitude of compressive and tensile loadings, and boundary conditions were all defined, and meshing of the models was performed. The von Mises stress, shear stress, deformation, and strain were calculated and recorded separately for each occlusal scheme of AGF, PGF, and CG.

**Table 1:** Mechanical properties of the components

Component	Young's modulus (MPa)	Poisson's ratio
**Cortical bone**	13,700	0.3
**Cancellous bone**	1,370	0.3
**Porcelain**	82,800	0.35
**PDL**	69	0.45
**Tooth**	18,000	0.33

Abbreviation: PDL, periodontal ligament.

In PGF, load was applied laterally to the middle and incisal thirds of the lingual surface of the maxillary canine and the palatal surface of the buccal cusps of the maxillary posterior teeth.

In AGF, load was applied laterally to the lingual surface of the maxillary canine and incisors.

In CG, load was applied to the palatal surface of the maxillary canine. Dynamic load was applied in all three occlusal schemes. Dynamic loads were applied in a two-step process following a time-dependent pattern. In both the vertical load step and the oblique load step, the loads exhibited a ramplike behavior, gradually increasing from 0 to 100 in accordance with the time-dependent characteristics.

**Fig. 1:**
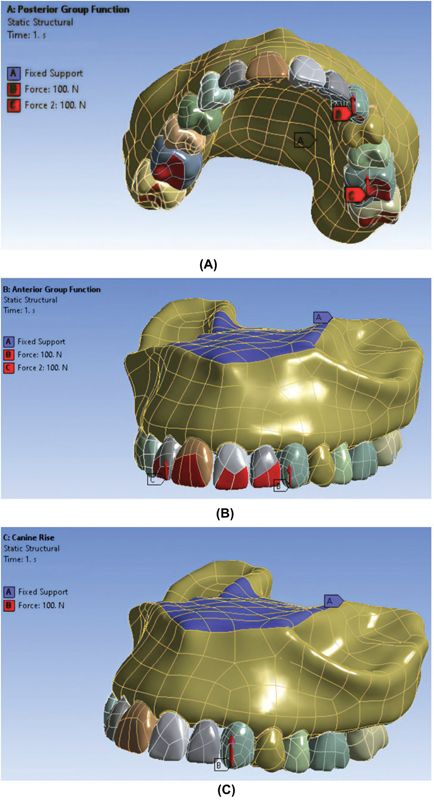
Loading and boundary condition in three occlusal schemes. ( **A** ) Canine guidance. ( **B** ) Anterior group function. ( **C** ) Posterior group function.

To enhance precision while dealing with dynamic loads, we employed C3D10 elements as our primary choice, complemented by C3D15 3D elements, both of which possess polyhedral characteristics. Also, the free mesh model was used due to complex boundaries of the model. Tiny elements were selected due to very low thickness of the implant layer. A 100-N load was applied to simulate functional loads in the three occlusal schemes, and the upper part of the maxilla was considered fixed, as illustrated in Fig. 1 . The total number of tetrahedral elements was 128,495 and the total number of nodes was 238,154.

Data were analyzed by ABAQUS software to calculate the stress transferred to the canine implant and the maxillary bone in the three occlusal schemes.

## Results

**Fig. 2:**
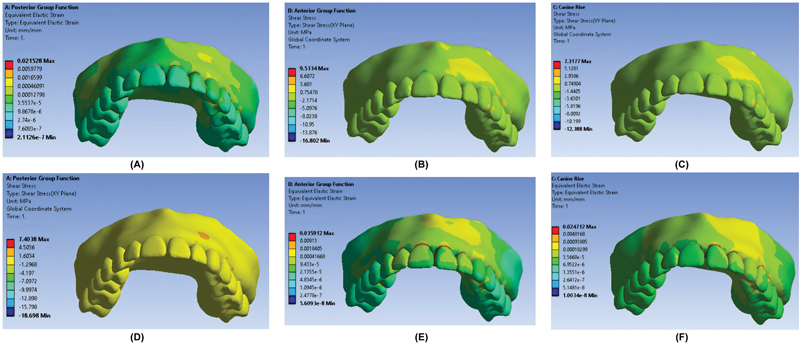
Strain and stress distribution. ( **A** ) Elastic strain in canine guidance occlusal scheme. ( **B** ) Elastic strain in anterior group function occlusal scheme. ( **C** ) Elastic strain in posterior group function occlusal scheme. ( **D** ) Shear stress in canine guidance occlusal scheme. ( **E** ) Shear stress in anterior group function occlusal scheme. ( **F** ) Shear stress in posterior group function occlusal scheme.

The maximum and minimum elastic strain and shear stress ( Fig. 2 ) values were noted in the AGF and PGF occlusal schemes, respectively, in all teeth.

**Fig. 3:**
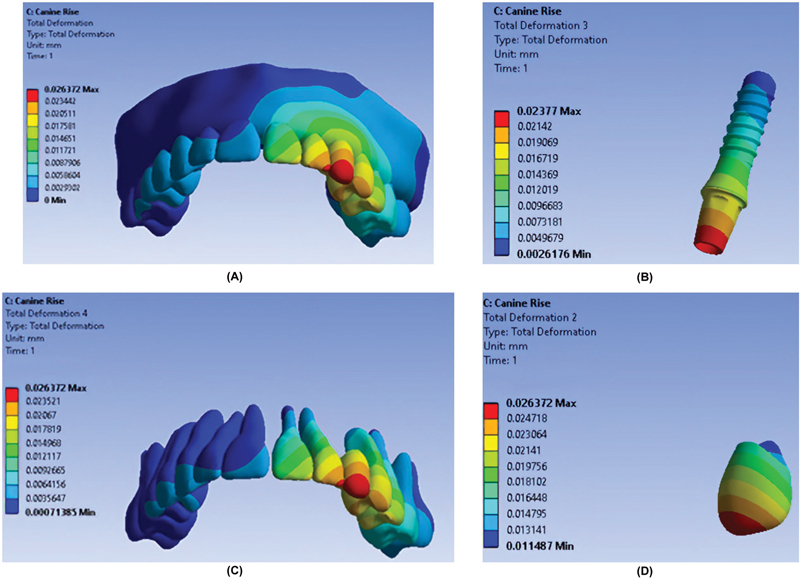
Total deformation of components in the canine guidance occlusal scheme. ( **A** ) Entire model. ( **B** ) Fixture. ( **C** ) Teeth. ( **D** ) Restoration.

**Fig. 4:**
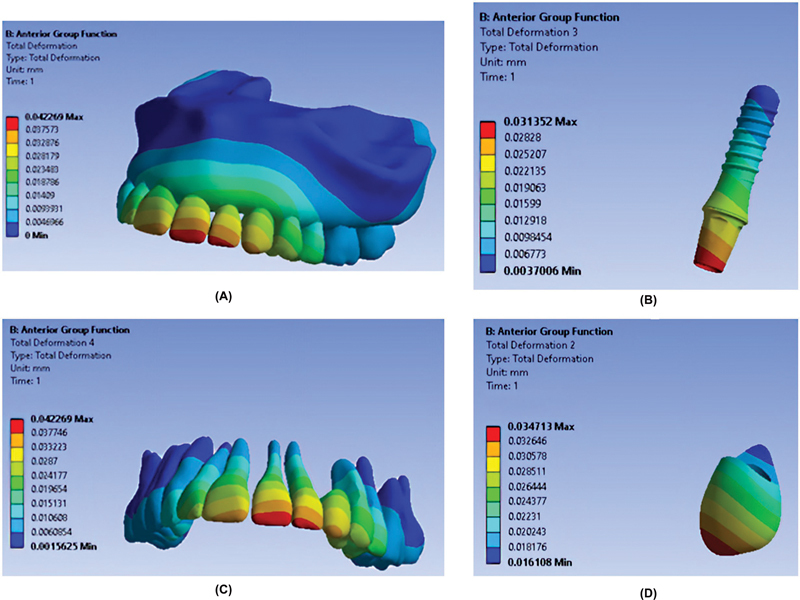
Total deformation of components in the anterior group function occlusal scheme. ( **A** ) Entire model. ( **B** ) Fixture. ( **C** ) Teeth. ( **D** ) Restoration.

**Fig. 5:**
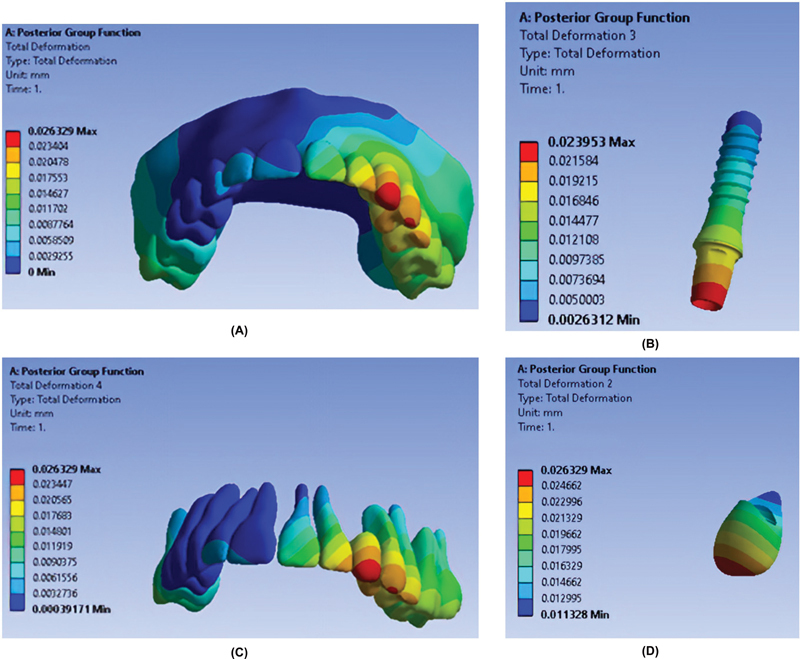
Total deformation of components in the posterior group function occlusal scheme. ( **A** ) Entire model. ( **B** ) Fixture. ( **C** ) Teeth. ( **D** ) Restoration.

As shown in Figs. 3 , 4 , 5 , the maximum total deformation in all teeth was recorded in the AGF occlusal scheme, and the minimum total deformation was seen in the PGF occlusal scheme.

The maximum level of von Mises stress in the CG scheme was recorded in the teeth adjacent to the canine tooth (first premolar and lateral incisor) and the lowest stress was recorded in the first and second molar teeth. Also, maximum elastic strain in CG was noted in the lateral and central incisors, the first and second premolars, and the first molar in the cervical region. The lowest elastic strain was the same in all teeth. Maximum shear stress in CG was noted in the lateral incisor, canine, and first premolar. The lowest stress was the same in all teeth. Regarding total deformation, maximum deformation was noted in the lateral incisors and the first premolars. Thus, maximum stress was recorded in teeth adjacent to the canine tooth.

In the AGF, the maximum von Mises stress was recorded in teeth adjacent to the canine tooth, such that the central and lateral incisors and the first and second premolars showed the highest stress. Also, the lowest stress was recorded in the occlusal three-fourths of the crown of the first molar and the entire second molar. The amount of elastic strain in the AGF was the same in all teeth, but the first premolar and first and second molars of the other side of the jaw showed minimum stress. Also, the maximum shear stress in the AGF was recorded in the lateral incisor, canine, and first premolar teeth. Regarding total deformation, maximum deformation was noted in the lateral and central incisors. Thus, maximum stress was recorded in the teeth adjacent to the canine tooth in the AGF.

In the PGF, the maximum von Mises stress was noted in the first premolars and lateral incisors. Also, elastic strain was higher in the first and second premolars than the other teeth, and was the same in the remaining teeth. Shear stress was the same in all teeth. Also, maximum deformation was noted in the first premolar.

Table 2 presents the von Mises stress values applied to maxillary canine implant restoration in different occlusal schemes. As shown, the maximum and minimum von Mises stress values in canine implant restoration were noted in the AGF and PGF, respectively. Moreover, Table 2 indicates the von Mises stress values applied to maxillary canine implant fixture in different occlusal schemes. As shown, the maximum and minimum von Mises stress values in maxillary canine implant fixture were noted in the AGF and PGF, respectively.

**Table 2:** von Mises stress values applied to maxillary canine implant restoration and fixture in different occlusal schemes (in MPa)

	Occlusal scheme	Maximum	Site of maximum stress accumulation	Minimum	Site of minimum stress accumulation
Restoration	Anterior group function	40.664	Cervical region, one point in the distal surface	1.144	Mesio-incisal angle
	Canine guidance	32.405	Cervical region, one point in the distal surface	0.59057	One point in the incisal edge
	Posterior group function	31.844	Cervical region, one point in the distal surface	0.32854	One point in the incisal edge
Fixture	Anterior group function	74.961	One point in the cervical part of fixture	0.746	Palatal surface of fixture
	Canine guidance	58.764	One point in the cervical part of fixture	0.19425	Palatal surface of fixture
	Posterior group function	58.7	One point in the cervical part of fixture	0.23437	Palatal surface of fixture

Table 3 presents the total deformation of restoration in different occlusal schemes. As indicated, the maximum and minimum total deformation of canine restoration occurred in the AGF and PGF, respectively. In addition, Table 3 indicates the total deformation of implant in different occlusal schemes. As indicated, the maximum and minimum total deformation of canine implant occurred in the AGF and PGF, respectively.

**Table 3:** Total deformation of restoration and implant in different occlusal schemes (in mm)

	Occlusal scheme	Maximum	Site of maximum deformation	Minimum	Site of minimum deformation
Restoration	Anterior group function	0.034713	Mesio-incisal angle of restoration	0.018176	Cervical part of the palatal restoration surface
	Posterior group function	0.026329	Incisal one-fifth of restoration	0.011328	Cervical part of the palatal restoration surface
	Canine guidance	0.026372	Incisal one-fifth of restoration	0.013141	Cervical part of palatal restoration surface
Implant	Anterior group function	0.031352	Incisal third of abutment	0.006773	Apical end of fixture
	Posterior group function	0.023953	Incisal third of abutment	0.0050003	Apical end of fixture
	Canine guidance	0.02377	Incisal third of abutment	0.0049679	Apical end of fixture

Regarding the peri-implant bone, the site of maximum stress accumulation in bone around the implant was the same in all occlusal schemes, and close to the implant neck.

Maximum total deformation in the peri-implant bone was recorded in the AGF, and minimum total deformation was noted in the PGF (with a small difference with CG). The maximum and minimum von Mises stress values the in peri-implant bone were recorded in the AGP and CG, respectively. The maximum and minimum shear stress values in the peri-implant bone were recorded in the PGF and CG, respectively. The maximum and minimum elastic strain in the peri-implant bone were recorded in the AGF and CG, respectively.

## Discussion

The occlusal scheme is an important factor that needs to be addressed to improve the prognosis of dental implant treatment. 14 15 FEA is a reliable method for assessment of stress and strain distribution patterns. 16 This study compared the stress and strain distribution patterns in canine implant and maxillary bone in the AGF, PGF, and CG occlusal schemes by FEA. FEA provides standard models, and has been previously used for assessment of different occlusal schemes. 17 The present results revealed a significant difference in the amount of stress applied to the maxillary canine implant and maxillary bone in the three occlusal schemes, and maximum stress was noted in the AGF, while minimum stress was recorded in the PGF scheme. The level of stress in the CG scheme was close to that in the PGF.

The maximum level of von Mises stress in the CG scheme was recorded in teeth adjacent to the canine tooth (first premolar and lateral incisor) and the lowest stress was recorded in the first and second molar teeth. In the AGF, the maximum von Mises stress was recorded in the teeth adjacent to the canine tooth, such that the central and lateral incisors and the first and second premolars showed the highest stress. The lowest stress was recorded in the occlusal three-fourths of the crown of the first molar and the entire second molar. In the PGF, the maximum von Mises stress was noted in the first premolars and lateral incisors.

The results of most previous studies on stress distribution in dental implant, tooth, and bone were in agreement with the present findings. Himmlová et al, 18 Anitua et al, 14 Pellizzer et al, 15 and Takahashi et al 19 reported maximum stress accumulation in the implant neck, and also at the framework–abutment interface, and the terminal end of framework. In the surrounding bone, maximum stress distribution is often in the cortical bone around the implant neck. However, some other studies reported different results. Hidaka et al 20 and Roque et al 21 reported maximum stress distribution in the posterior region. Difference between the present results and those of Hidaka et al 20 and Roque et al 21 can be due to insertion of posterior implants close to the hinge axis of the jaws. Thus, in designing implant-supported fixed partial dentures, the amount of load applied to tooth, bone, and implant should be considered. Bone quality should be well assessed while selecting the treatment type, implant type, and implant site. The implant system used in the posterior region should well resist the masticatory forces. 17 Türker et al 17 evaluated the amount of load applied to bone and implant in different occlusal schemes and reported that the CG applies lower stress than the AGF and PGF. In the present study, the PGF applied minimum stress to canine implant and maxillary bone, and the AGF resulted in maximum stress accumulation in canine implant and maxillary bone. Thus, their results were different from the present findings, which may be due to the difference in the study design and methodology.

In this study, we have meticulously evaluated the mechanical parameters (stress and strain) for all model components, ensuring none of them exhibit yielding behavior. Nevertheless, we propose that in future research the PDL be examined using viscoelastic and hyperelastic materials to enhance result precision when compared with the use of elastic materials. Furthermore, we recommend parallel clinical assessments in future investigations to validate numerical outputs from the software by comparing them with experimental data, thus confirming the credibility of our simulation results.

## Conclusion

The PGF showed minimum von Mises stress, elastic strain, shear stress, and deformation in canine implant and maxillary bone. Thus, it appears than the PGF is the best occlusal scheme for maxillary canine implant followed by the CG scheme.

## References

[1] Pjetursson B E Thoma D Jung R Zwahlen M Zembic A A systematic review of the survival and complication rates of implant-supported fixed dental prostheses (FDPs) after a mean observation period of at least 5 years Clin Oral Implants Res 2012 23 06 22 38 10.1111/j.1600-0501.2012.02546.x 23062125

[2] Mazaro J V Filho H G Vedovatto E Pellizzer E P Rezende M C Zavanelli A C Evaluation of stress patterns produced by implant-retained overdentures and implant-retained fixed partial denture J Craniofac Surg 2011 22 06 2153 2157 22067869 10.1097/SCS.0b013e3182323e29

[3] Papaspyridakos P Chen C J Singh M Weber H P Gallucci G O Success criteria in implant dentistry: a systematic review J Dent Res 2012 91 03 242 248 22157097 10.1177/0022034511431252

[4] Lops D Bressan E Chiapasco M Rossi A Romeo E Zirconia and titanium implant abutments for single-tooth implant prostheses after 5 years of function in posterior regions Int J Oral Maxillofac Implants 2013 28 01 281 287 23377075 10.11607/jomi.2668

[5] Frizzera F Oliveira G JPL Shibli J A Moraes K C Marcantonio E B Marcantonio Junior E Treatment of peri-implant soft tissue defects: a narrative review Braz Oral Res 2019 33 01 e073 31576957 10.1590/1807-3107bor-2019.vol33.0073

[6] Koller C D Pereira-Cenci T Boscato N Parameters associated with marginal bone loss around implant after prosthetic loading Braz Dent J 2016 27 03 292 297 27224562 10.1590/0103-6440201600874

[7] Sheridan R A Decker A M Plonka A B Wang H L The role of occlusion in implant therapy: a comprehensive updated review Implant Dent 2016 25 06 829 838 27749518 10.1097/ID.0000000000000488

[8] Kato H Kuroshima S Inaba N Uto Y Sawase T Effect on bone architecture of marginal grooves in dental implants under occlusal loaded conditions in beagle dogs J Oral Implantol 2018 44 01 37 45 29135387 10.1563/aaid-joi-D-17-00020

[9] Ün M Çalık A Relevance of inhomogeneous–anisotropic models of human cortical bone: a tibia study using the finite element method Biotechnol Biotechnology Equip 2016 30 538 547

[10] Heintze S D Monreal D Reinhardt M Fatigue resistance of all-ceramic fixed partial dentures: fatigue tests and finite element analysis Dent Mater 2018 34 03 494 507 29395474 10.1016/j.dental.2017.12.005

[11] Yoda N Liao Z Chen J Sasaki K Swain M Li Q Role of implant configurations supporting three-unit fixed partial denture on mandibular bone response: biological-data-based finite element study J Oral Rehabil 2016 43 09 692 701 27224022 10.1111/joor.12411

[12] Modi R Kohli S Rajeshwari K Bhatia S A three-dimension finite element analysis to evaluate the stress distribution in tooth supported 5-unit intermediate abutment prosthesis with rigid and nonrigid connector Eur J Dent 2015 9 02 255 261 26038660 10.4103/1305-7456.156847 PMC4439856

[13] Burak Özcelik T Ersoy E Yilmaz B Biomechanical evaluation of tooth- and implant-supported fixed dental prostheses with various nonrigid connector positions: a finite element analysis J Prosthodont 2011 20 01 16 28 21251117 10.1111/j.1532-849X.2010.00654.x

[14] Anitua E Tapia R Luzuriaga F Orive G Influence of implant length, diameter, and geometry on stress distribution: a finite element analysis Int J Periodontics Restorative Dent 2010 30 01 89 95 20224835

[15] Pellizzer E P Verri F R de Moraes S L Falcón-Antenucci R M de Carvalho P S Noritomi P Y Influence of the implant diameter with different sizes of hexagon: analysis by 3-dimensional finite element method J Oral Implantol 2013 39 04 425 431 21463183 10.1563/AAID-JOI-D-10-00103

[16] Van Staden R C Guan H Loo Y C Application of the finite element method in dental implant research Comput Methods Biomech Biomed Engin 2006 9 04 257 270 17132532 10.1080/10255840600837074

[17] Türker N Büyükkaplan U S Sadowsky S J Özarslan M M Finite element stress analysis of applied forces to implants and supporting tissues using the “all-on-four” concept with different occlusal schemes J Prosthodont 2019 28 02 185 194 30515911 10.1111/jopr.13004

[18] Himmlová L Dostálová T Kácovský A Konvicková S Influence of implant length and diameter on stress distribution: a finite element analysis J Prosthet Dent 2004 91 01 20 25 14739889 10.1016/j.prosdent.2003.08.008

[19] Takahashi J M Dayrell A C Consani R L de Arruda Nóbilo M A Henriques G E Mesquita M F Stress evaluation of implant-abutment connections under different loading conditions: a 3D finite element study J Oral Implantol 2015 41 02 133 137 23574455 10.1563/AAID-JOI-D-11-00205

[20] Hidaka O Iwasaki M Saito M Morimoto T Influence of clenching intensity on bite force balance, occlusal contact area, and average bite pressure J Dent Res 1999 78 07 1336 1344 10403461 10.1177/00220345990780070801

[21] Roque M A Gallucci G O Lee S J Occlusal pressure redistribution with single implant restorations J Prosthodont 2017 26 04 275 279 27706865 10.1111/jopr.12552

